# *Ex Vivo* Host and Parasite Response to Antileishmanial Drugs and Immunomodulators

**DOI:** 10.1371/journal.pntd.0003820

**Published:** 2015-05-29

**Authors:** Laura Gonzalez-Fajardo, Olga Lucía Fernández, Diane McMahon-Pratt, Nancy Gore Saravia

**Affiliations:** 1 Centro Internacional de Entrenamiento e Investigaciones Médicas (CIDEIM), Cali, Colombia; 2 Yale University School of Public Health, New Haven, Connecticut, United States of America; Universidade Federal de Minas Gerais, BRAZIL

## Abstract

**Background:**

Therapeutic response in infectious disease involves host as well as microbial determinants. Because the immune and inflammatory response to *Leishmania* (*Viannia*) species defines the outcome of infection and efficacy of treatment, immunomodulation is considered a promising therapeutic strategy. However, since *Leishmania* infection and antileishmanial drugs can themselves modulate drug transport, metabolism and/or immune responses, immunotherapeutic approaches require integrated assessment of host and parasite responses.

**Methodology:**

To achieve an integrated assessment of current and innovative therapeutic strategies, we determined host and parasite responses to miltefosine and meglumine antimoniate alone and in combination with pentoxifylline or CpG 2006 in peripheral blood mononuclear cells (PBMCs) of cutaneous leishmaniasis patients. Parasite survival and secretion of TNF-α, IFN-γ, IL-10 and IL-13 were evaluated concomitantly in PBMCs infected with *Luc-L*. (*V*.) *panamensis* exposed to meglumine antimoniate (4, 8, 16, 32 and 64 μg Sb^V^/mL) or miltefosine (2, 4, 8, 16 and 32 μM HePC). Concentrations of 4 μM of miltefosine and 8 μg Sb^V^/mL were selected for evaluation in combination with immunomodulators based on the high but partial reduction of parasite burden by these antileishmanial concentrations without affecting cytokine secretion of infected PBMCs. Intracellular parasite survival was determined by luminometry and cytokine secretion measured by ELISA and multiplex assays.

**Principal Findings:**

Anti- and pro-inflammatory cytokines characteristic of *L*. (*V*.) *panamensis* infection were evaluable concomitantly with viability of *Leishmania* within monocyte-derived macrophages present in PBMC cultures. Both antileishmanial drugs reduced the parasite load of macrophages; miltefosine also suppressed IL-10 and IL-13 secretion in a dose dependent manner. Pentoxifylline did not affect parasite survival or alter antileishmanial effects of miltefosine or meglumine antimoniate. However, pentoxifylline diminished secretion of TNF-α, IFN-γ and IL-13, cytokines associated with the outcome of infection by species of the *Viannia* subgenus. Exposure to CpG diminished the leishmanicidal effect of meglumine antimoniate, but not miltefosine, and significantly reduced secretion of IL -10, alone and in combination with either antileishmanial drug. IL-13 increased in response to CpG plus miltefosine.

**Conclusions and Significance:**

Human PBMCs allow integrated *ex vivo* assessment of antileishmanial treatments, providing information on host and parasite determinants of therapeutic response that may be used to tailor therapeutic strategies to optimize clinical resolution.

## Author Summary

Host determinants of the response to infection have increasingly been recognized as therapeutically relevant targets. Despite the pathogenesis of dermal leishmaniasis being mediated by the immune and inflammatory response, *in vitro* anti-leishmanial drug screening has been based on antimicrobial effect without consideration of effects on the host response. The results of this study show that peripheral blood mononuclear cells from patients allow an integrated evaluation of both antimicrobial efficacy and host response to drugs, immunomodulatory agents, and their combinations. This integrated approach to defining treatment strategies based on host and parasite responses opens the way for the optimization and tailoring of treatment to different clinical circumstances.

## Introduction

The outcome of treatment of leishmaniasis and other infectious diseases is multi-factorial involving host as well as microbial determinants; yet evaluation of antimicrobial drug susceptibility is limited to assessment of drug effects on microbial pathogens and toxicity. However, the efficacy of antimicrobial treatment is linked to the immunocompetence of the host [1–3], and to the role of host defense mechanisms in the containment of infection or pathogenesis of disease [4]. Hence *in vitro* assay systems of antimicrobial drug susceptibility that allow characterization of host response as well as antimicrobial effect are an important unmet need. The immune and inflammatory responses induced by infection with species of the *Leishmania* (*Viannia*) subgenus are pivotal in the pathogenesis of cutaneous and mucosal disease [5–7] and immune competence influences the efficacy of treatment [8]. These findings have motivated the exploration of therapeutic vaccines and immunomodulators as treatment strategies.

*Leishmania* are obligate intracellular pathogens that modulate a diverse range of host cell functions [9, 10] including the expression and function of drug transporters and metabolizing enzymes [11–13] and innate immune mechanisms [14, 15]. The capacity of *Leishmania* to modify and to effectively subvert host cell functions in favor of their survival and persistence constitutes a further and complex challenge to therapeutic interventions. Although specific parameters of the host response have been evaluated independently of anti-parasite effects for a few individual drugs [16–18], *ex vivo* surrogates of therapeutic response are critical to the development and preclinical evaluation of more effective treatments.

Pentoxifylline is thought to mediate immunomodulatory effects via inhibition of the synthesis of TNF-α [19] and has been used in combination with meglumine antimoniate to achieve healing of refractory mucosal leishmaniasis, a disease presentation associated with an intense inflammatory response. Addition of pentoxifylline increased the cure rate and decreased the plasma levels of inflammatory cytokines such as IFN-γ and TNF-α [20, 21]. Responses to CpG ODN have been found to be dose-dependent and of either Th1-type or T-regulatory-type in mouse models and humans [22–25] and have been investigated in human clinical trials for allergy, cancer, and autoimmunity [26, 27]. The combination of CpG with miltefosine has been evaluated in the treatment of experimental visceral leishmaniasis allowing reduction of both the duration and dosage of miltefosine [28]. Infected hamsters and mice that received the combination therapy presented significantly suppressed levels of Th2 cytokines (IL-10 and TGF-β) and increased mRNA expression levels of pro-inflammatory cytokines (IFN-γ, TNF-α and IL-12) [28].

This study reports the development of an *ex vivo* model of human host and parasite responses to antileishmanial drugs, immunomodulators and their combinations based on PBMCs from cutaneous leishmaniasis patients.

## Methods

### Study Design

In order to discern the anti-parasitic and immunomodulatory effects of anti-leishmanial therapies, we have developed an *ex vivo* model that allows concomitant quantitative evaluation of parasite survival and the elicited immune response within the context of active human leishmaniasis. Human PBMC cultures are widely utilized as a surrogate of the cell-mediated host response. These cultures, in addition to T and B lymphocytes and NK cells, contain monocytes that can differentiate to macrophages, which are the natural host cell of *Leishmania*. We therefore determined the culture conditions, period of exposure and concentration of anti-leishmanial drugs and immunomodulators alone or in combination that allowed the concomitant evaluation of intracellular parasite survival and the secretion of the pro- and anti-inflammatory cytokines IFN-γ, TNF-α, IL-13 and IL-10 induced by *Leishmania* infection of PBMCs from patients having active cutaneous leishmaniasis. The host and parasite response to antileishmanial drugs meglumine antimoniate and miltefosine, which are widely used for the routine treatment of dermal leishmaniasis in the Americas, were evaluated individually and in combination with the immunomodulators pentoxifylline and CpG ODN 2006.

### Study Population

Overall twenty-two male and female patients, 18 years of age or older, with parasitologically confirmed cutaneous leishmaniasis participated in the study. The number of participants in the various analyses was based on the sample size that in previous studies of the immune response in leishmaniasis patients has allowed significant differences in cytokine production to be detected [29]. Participants were from the Pacific coast region of Colombia and consulted the CIDEIM facilities in the municipality of Tumaco or Cali. All patients were enrolled within 5 months of the onset of disease (Mean 2.3 months) and before initiating treatment. Parasites isolated from the participants were principally *L*. *(V) panamensis* (18/19, 95%). All participants of the study were seronegative for HIV-1/HIV-2 by ELISA (Abbott Laboratories). Parasitological confirmation of leishmaniasis was based on detection of amastigotes in smears from the lesions and/or isolation and identification of the *Leishmania* cultured from tissue fluid aspirated from lesions.

### Ethics Statement

The study was approved and monitored by the CIDEIM Institutional Review Board for research involving human subjects in accordance with national and international guidelines for Good Clinical Practice. Voluntary, informed, signed consent was provided by each participant.

### Anti-leishmanial Drugs and Immunomodulators

Stock solutions of meglumine antimoniate (Sb^V^) (Walter Reed 214975AK; lot no. BLO9186 90-278-1A1 W601; antimony analysis, 25%–26.5% by weight) (30 mg/mL); pentoxifylline (PTX) (P1784; Sigma) (10 mM), and CpG ODN 2006 (tlrl-2006; Invitrogen) (500 μM) were prepared by dissolution in sterile water, filtered through a 0.22 μm membrane (MS MCE Syringe filters; Membrane Solutions) and stored at -20°C. Based on solubility characteristics, miltefosine (HePC) (63280; Cayman Chemical Co) was dissolved in sterile dimethyl sulfoxide at a concentration of 1.96 mM and stored at -20°C until use. Dilutions of the drugs were prepared in RPMI 1640 medium (Sigma) containing 10% heat inactivated fetal bovine serum (FBS) on the day of use.

### PBMC Isolation

Blood samples of 100 mL were collected after confirming diagnosis and before initiation of treatment. PBMCs were isolated by centrifugation over Histopaque 1077 solution (Sigma-Aldrich) according to the product instructions. The cells were frozen in 90% FBS plus 10% DMSO by slow cooling at approximately 1°C/minute using a freezing container (Thermo Scientific) and stored in liquid nitrogen until the time of experimental evaluation. Prior to each experiment, the cells were rapidly thawed at 37°C and PBMCs with ≥ 90% viability were used for the experiments [30].

### Drug Cytotoxicity Control for PBMCs

To control potential confounding effects of drug cytoxicity for host PBMCs, viability was evaluated based on acid phosphatase activity after 120 h exposure to meglumine antimoniate, miltefosine and pentoxifylline at the concentrations employed in the combinations of drugs and immunomodulators [31]. No significant reduction in cell viability was observed when comparing treated cells vs non-treated controls. Previous studies have demonstrated that CpG, even at high concentrations (6 μM), does not alter the viability of human cells [32, 33].

### Standardization of PBMC Infection for Detection of Parasite Burden and Cytokine Secretion

For all experiments PBMCs were cultured at a final concentration of 2 x 10^6^ mL. Thawed PBMCs were resuspended at 4x10^6^ cells/mL RPMI-1640 medium supplemented with heat inactivated 10% FBS (complete medium), dispensed as 100 μL aliquots (4x10^5^ cells/well) in 96-well plates and cultured for 2 h at 37°C and 5% CO_2_ to initiate adherence. Infection with *L*. *(Viannia) panamensis* promastigotes transfected with the luciferase reporter gene (*Luc*), MHOM/COL/03/3594/LUC001 [34, 35] was achieved by adding 50 μL of opsonized stationary phase promastigotes in complete medium at 20:1 or 10:1 parasite to monocyte ratios and incubation for 24 hours at 34°C. Fifty μL of complete culture medium were then added to infected PBMCs. Parasite burden and Th1/Th2 cytokine secretion were evaluated after 6, 12, 24, 48 and 72 h incubation at 34°C, 5% CO_2_.

Prior to infection, parasites were opsonized for 1 h at 34°C in RPMI 1640 containing 10% heat-inactivated AB+ human serum [36]. In order to standardize the inoculum for PBMCs, the parasite concentration to achieve the corresponding parasite to monocyte ratio was operationally defined based on the assumption that monocytes obtained from heparinized whole blood and multiple washings constituted approximately 10% of the mononuclear cells [37]. To evaluate and confirm the infection of macrophages differentiated from monocytes in PBMC cultures, parasite burden in total PBMCs was compared with that of adherent cells alone, using an infection ratio of 10:1. For this assessment, luminometric readout of parasite burden was conducted in parallel in PBMCs and after removal of non-adherent cells and extracellular parasites, and the internalization of parasites was confirmed by microscopy”. Responses of PBMCs from individual patients were evaluated in triplicate.

### Dose-Response and Kinetics Assays of Anti-leishmanial Drugs

Dose-response and kinetics assays of miltefosine and meglumine antimoniate were conducted to determine their effect on TNF-α, IFN-γ, IL-13 and IL-10 secretion and parasite burden and the relationship between these parameters. Antileishmanial drugs were added to PBMCs infected for 24 h with Luc-*L*. *(V) panamensis*, at a parasite to monocyte ratio of 10:1. Drug concentrations evaluated were 4, 8, 16, 32 and 64 μg Sb^V^/mL as meglumine antimoniate and 2, 4, 8, 16 and 32 μM of miltefosine. This range was employed to determine the drug concentration that substantially reduced but did not eliminate infection (mean approximating 80%) compared with untreated controls, so that the effect of immunomodulators on parasite survival could be determined when combined with anti-leishmanial drugs. Kinetic assays were conducted at 24 hour intervals over 96 h after adding 4 μM of miltefosine or 8 μg Sb^V^/mL as meglumine antimoniate. These assays were conducted at 34°C, 5% CO_2_

### Evaluation of Response to Anti-lieshmanial Drugs and Immunomodulators

A range of concentrations of pentoxifylline and CpG 2006 were evaluated alone or in combination with 4 μM of miltefosine or 8 μg Sb^V^/mL in infected PBMCs. Evaluation of parasite survival and Th1/Th2 cytokine response was conducted with a parasite to monocyte ratio of 10:1 because the parasite burden and kinetics of parasite survival allowed dose-response analyses and discrimination of treated and untreated cultures. Immunomodulators were added at concomitantly with promastigotes to achieve concentrations of 100, 200 and 300 μM pentoxifylline and 2.5, 5, 10 μM CpG ODN 2006; anti-leishmanial drugs were added 24 h after infection. The concentration ranges of immunomodulators were based on prior exploratory experiments to evaluate the effect of pentoxifylline on TNF-α secretion and, previous investigations of CpG ODN 2006 in the *in vitro* immune response of human PBMCs [32, 33]. Parasite burden and cytokine secretion were evaluated at 120 h of culture, and 96 h after addition of anti-leishmanial drugs.

### Parasite and Cytokine Quantification

Plates were centrifuged at room temperature at 1097Xg for 10 min; supernatants were collected and stored at -80°C. Infection was quantified as luciferase activity using luminometry (Chameleon V Multilabel Microplater Reader; Hidex, Finland) as previously described [34]. Concordance between luminometric and conventional microscopic quantification of intracellular amastigotes has been previously reported for evaluation susceptibility to meglumine antimoniate and miltefosine [38, 39]. In the present study the limit of detection of the strain Luc-*L*. *(V) panamensis* (Luc 001) by luminometry was 50 promastigotes and 250 intracellular amastigotes. TNF-α, IL-10, IFN-γ, IL-13 were measured in supernatants by ELISA [40] or Luminex Screening Assay (R&D Systems, Minneapolis, MN, USA). Luminex assays were performed using 50 μL of culture supernatants in duplicate according to the manufacturers’ specifications.

### Statistical Analysis

One-way ANOVA or the Kruskal-Wallis tests were used to establish statistical differences among groups. Dunnett or Dunn tests were performed to compare each group with the control group, according to the parametric or non-parametric distribution of data. Analyses were performed with GraphPad Prism 6 software (GraphPad Inc., San Diego, CA), and P values < 0.05 were considered significant.

## Results

### Infection and Immune Response in the Absence of Drugs or Immunomodulators

**Fig 1:**
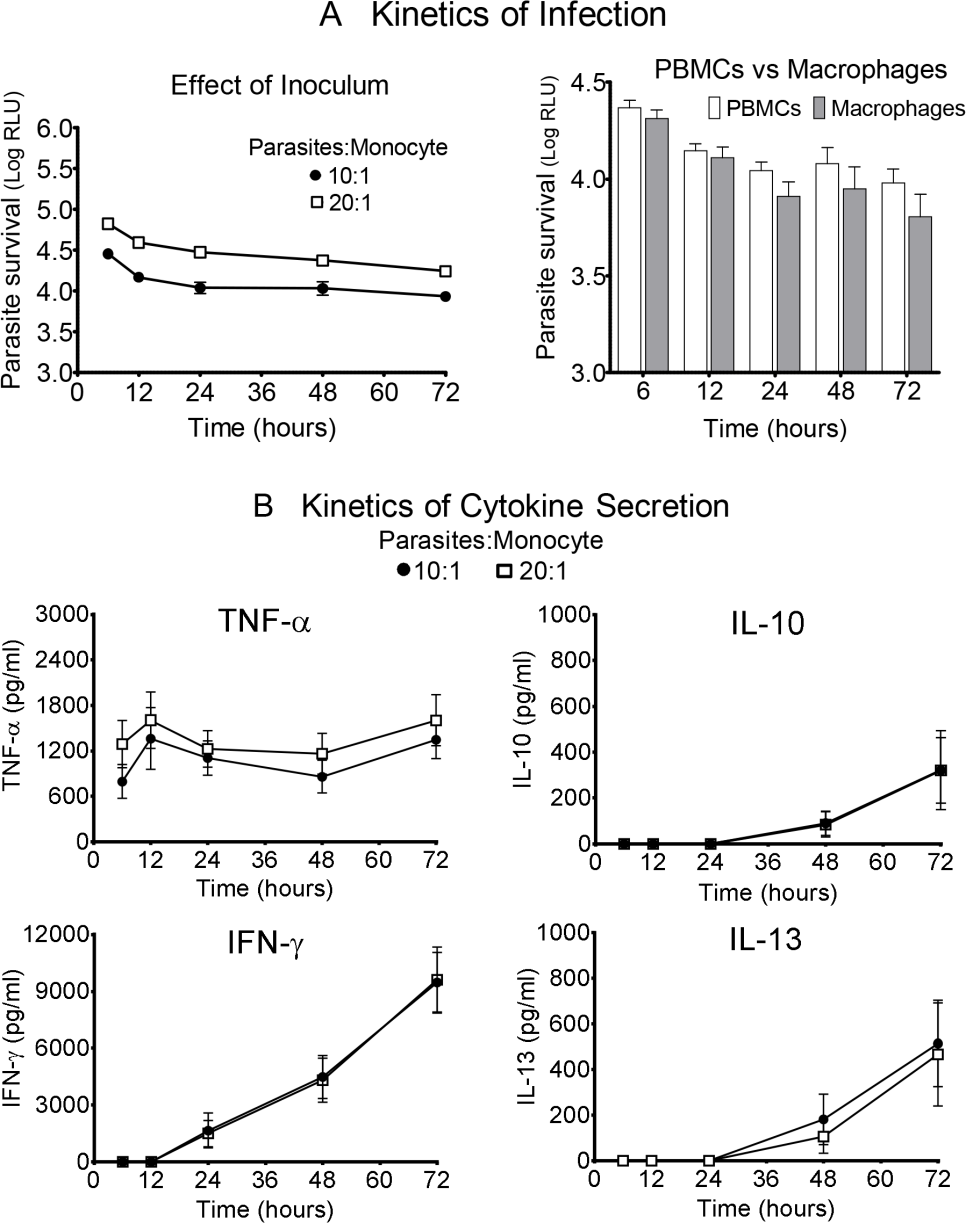
Kinetics of parasite and immunologic responses in the absence of drugs. (A) Kinetics of infection evaluating the parasites: monocyte ratios of 10:1 and 20:1 (left panel), and the parasite burden (right panel) in PBMCs vs macrophages alone (infection ratio 10:1) are shown. Parasite survival is expressed as bioluminescence produced by luciferase activity in relative light units (RLU). Mean signal of uninfected cells: 135.3 ± 23.7 RLU. (B) Kinetics of cytokine secretion (TNF-α, IL-10, IL-13, IFN-γ) over the 72 hours of treatment. Data are based on at least 4 patients and expressed as means ± SEM.

Parasite burden was proportional to the infective dose of parasites and measurable throughout the observation period of 6 to 72 hours post-infection (Fig 1A). The highest number of parasites was detected at the initial measurement 6 h post-infection and decreased thereafter. No significant differences were observed in the parasite burden in total PBMCs compared to macrophages alone (Fig 1A). Pro-inflammatory, anti-inflammatory and Th1/Th2 cytokines observed across the clinical spectrum of infection by *L*. *(V) panamensis* were induced under these experimental conditions and measured concurrently with the burden of infection. Kinetics of production of individual cytokines varied: TNF-α was secreted early after infection while other cytokines (IL-10, IL-13, IFN-γ) became detectable 24 to 48 hours post-infection (Fig 1B). Unlike parasite burden, cytokine expression was not proportional to the infective dose.

### Parasitological and Immunological Responses to Anti-leishmanial Drugs

**Fig 2:**
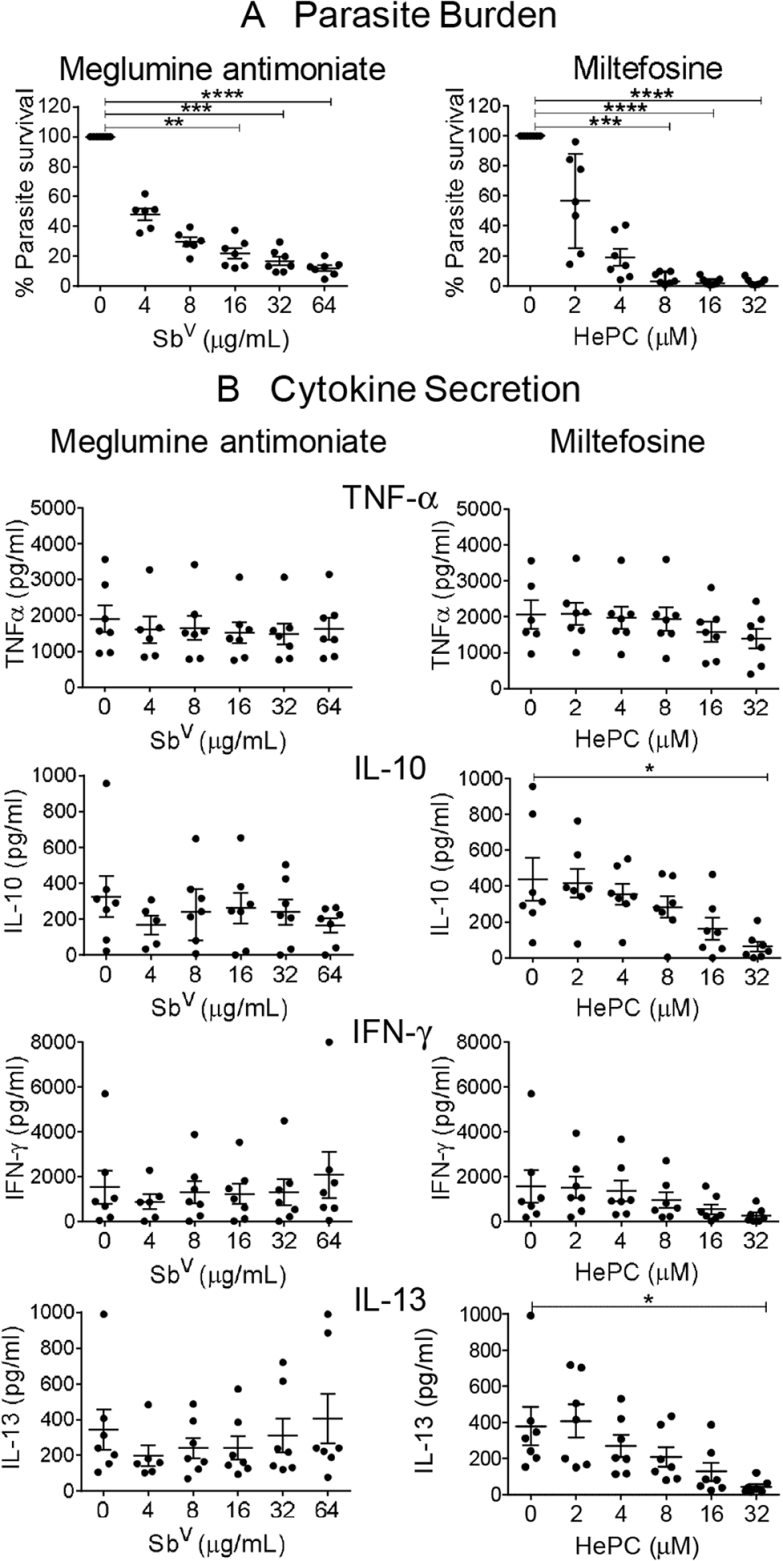
Concentration-dependent effect of miltefosine and meglumine antimoniate on parasite survival and cytokine secretion. (A) Parasite survival and (B) Cytokine secretion after 96 hours of exposure to increasing concentrations of miltefosine (HePC) and meglumine antimoniate (Sb^V^). TNFα, IL-10, IFNγ and IL-13 were evaluated in supernatants of PBMCs infected with *L*. *(V) panamensis*. Data are based on at least 6 patients and presented as mean ± SEM of the parasite burden or cytokine secretion compared to infected control without drug. ** p ≤ 0.01, *** p ≤ 0.001, **** p ≤ 0.0001.

Meglumine antimoniate and miltefosine reduced the parasite load in a concentration-dependent manner reaching statistical significance at the higher range of drug concentrations compared to untreated infected control PBMCs cultures (Fig 2A). Parasitological response was based on % reduction of signal compared with untreated PBMCs for each donor, thereby controlling for variation in parasite burden among donors. Miltefosine suppressed the production of IL-10 and IL-13 in a dose-dependent manner, with the decrease becoming statistically significant at 32 μM of drug compared to control (Fig 2B). In contrast, no significant effect on the production of IFN-γ or TNF-α was observed over the dose range evaluated for this drug. Notably meglumine antimoniate demonstrated statistically significant parasite reduction over a concentration range of 16–64 μg Sb^V^/ml but did not alter secretion of any of the four cytokines evaluated (Fig 2B). Based on the reduction of infection approximating 80% and non-inhibition of cytokine secretion of cytokines at the respective drug concentrations, 8 μg Sb^V^/mL of meglumine antimoniate and 4 μM of miltefosine were selected for the concomitant assay of parasite survival and immune response.

### Kinetics of Parasite Survival and Cytokine Production in the Presence of 4 μM of Miltefosine or 8 μg Sb^V^/mL of Meglumine Antimoniate

**Fig 3:**
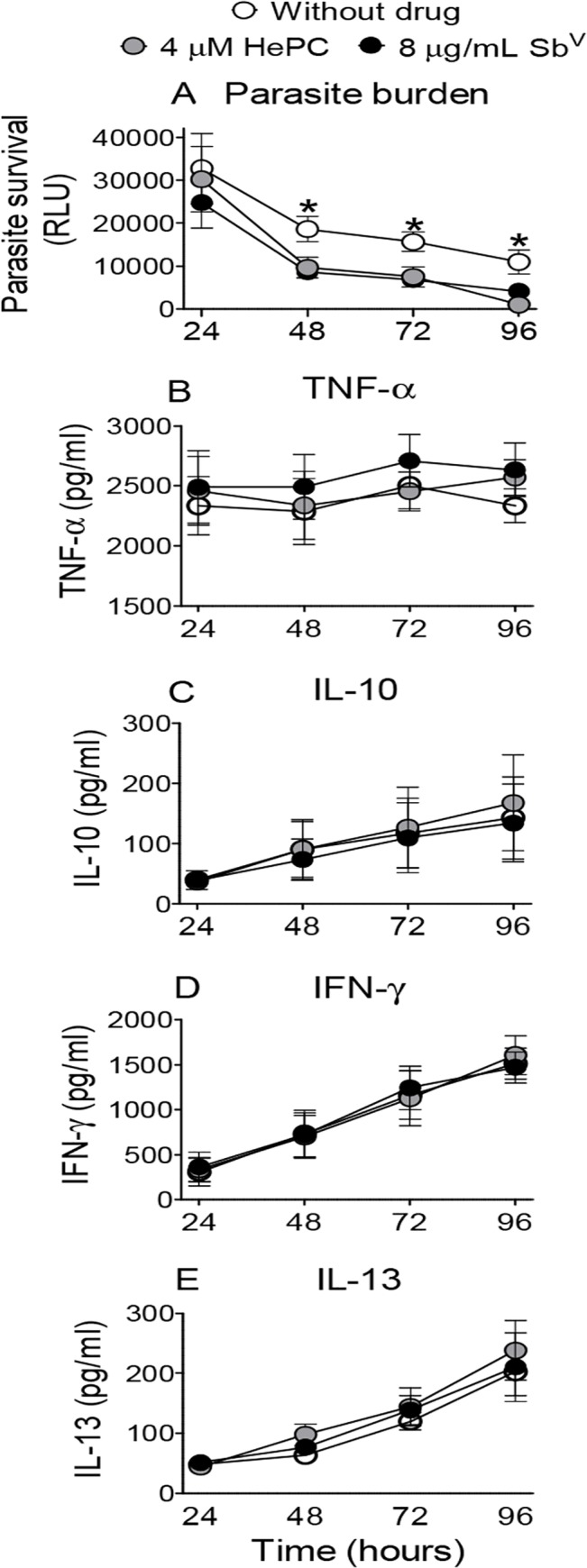
Kinetics of parasite survival and cytokine secretion in response to 4 μM miltefosine and 8 μg Sb^V^/mL as meglumine antimoniate in the *ex vivo* PBMC model. (A) Parasite survival and (B to E) cytokine secretion (IL-13, TNF-α, IL-10, IFN-γ) over 96 h. Mean values ± SEM for PBMCs from 5 patients. ** p ≤ 0.01, control vs both drugs.

Parasite burden was significantly reduced by 48 hours of exposure to 4 μM of miltefosine or 8 μg Sb^V^/mL of meglumine antimoniate. Survival continued to decline significantly reaching 9% and 37% at 96 hours at these concentrations of miltefosine and meglumine antimoniate respectively, compared with infection in the absence of drug (Fig 3A). Secretion of TNF-α, IL-10, IFN-γ and IL-13 was not affected by either drug at the concentrations evaluated (Fig 3B–3E).

### Parasitological and Immunological Responses to Combinations of Immunomodulators and Anti-leishmanial Drugs

**Fig 4:**
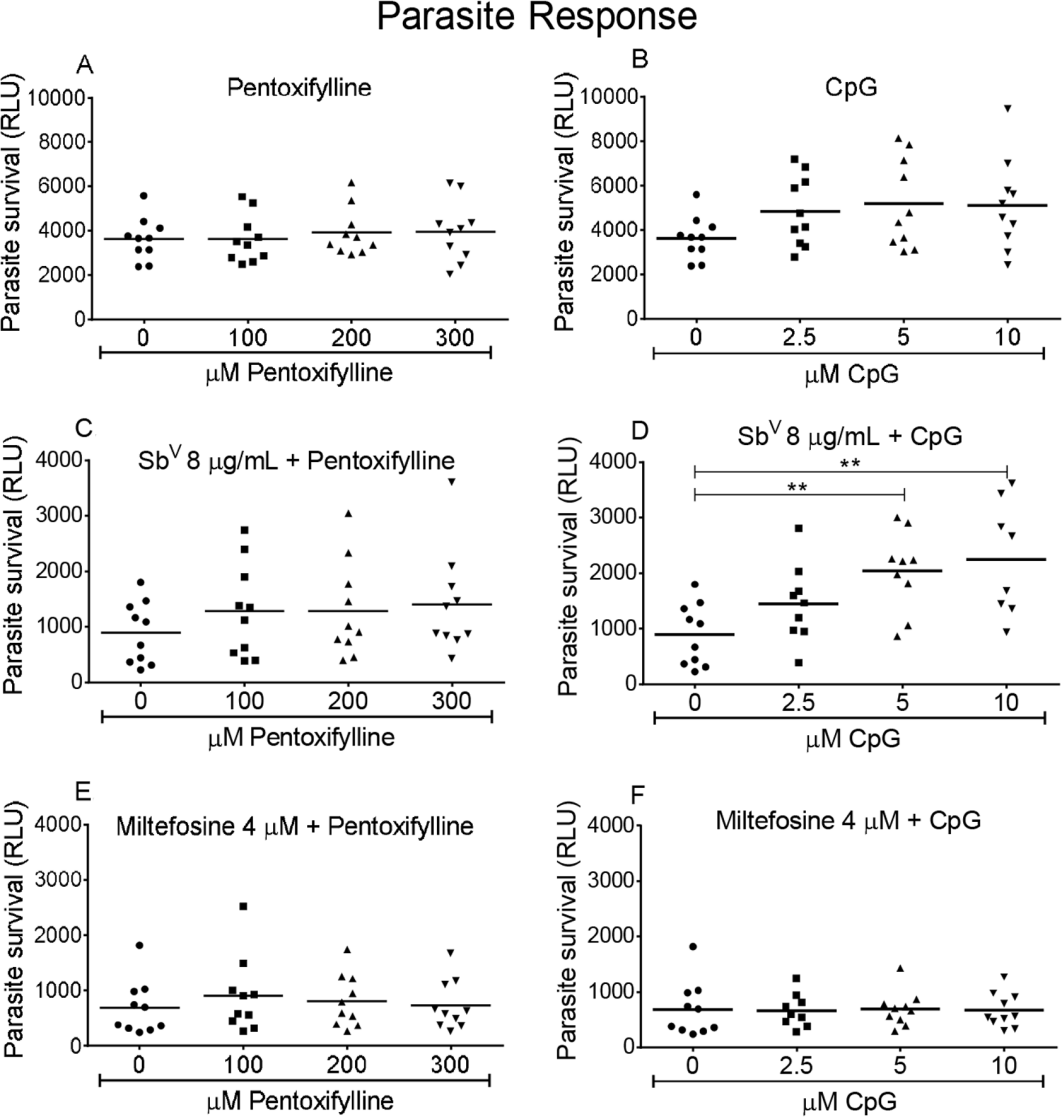
Effect of anti-leishmanial drugs, immunomodulators and their combinations on parasite burden. (A, C and E) Dose response for pentoxifylline alone, combined with 8 μg Sb^V^/ml meglumine antimoniate or 4 μM miltefosine. (B, D and F) Dose response for CpG alone, combined with 8 μg Sb^V^/ml meglumine antimoniate or 4 μM miltefosine. Data are presented as means ± SEM of the parasite burden compared to infected control cultures without drugs for PBMCs from 10 patients. * p< 0.05.

Parasite survival and cytokine production were differentially affected by the individual drugs and immunomodulators and their combinations. Pentoxifylline did not directly affect parasite survival (Fig 4A) or the leishmanicidal activity of the anti-leishmanial drugs evaluated (Fig 4C and 4E). CpG alone did not significantly affect parasite survival (Fig 4B) but its combination with meglumine antimoniate resulted in significantly lower leishmanicidal activity compared with antimonial drug alone (Fig 4D). In contrast, miltefosine-induced killing of parasites was not altered by CpG (Fig 4F).

**Fig 5:**
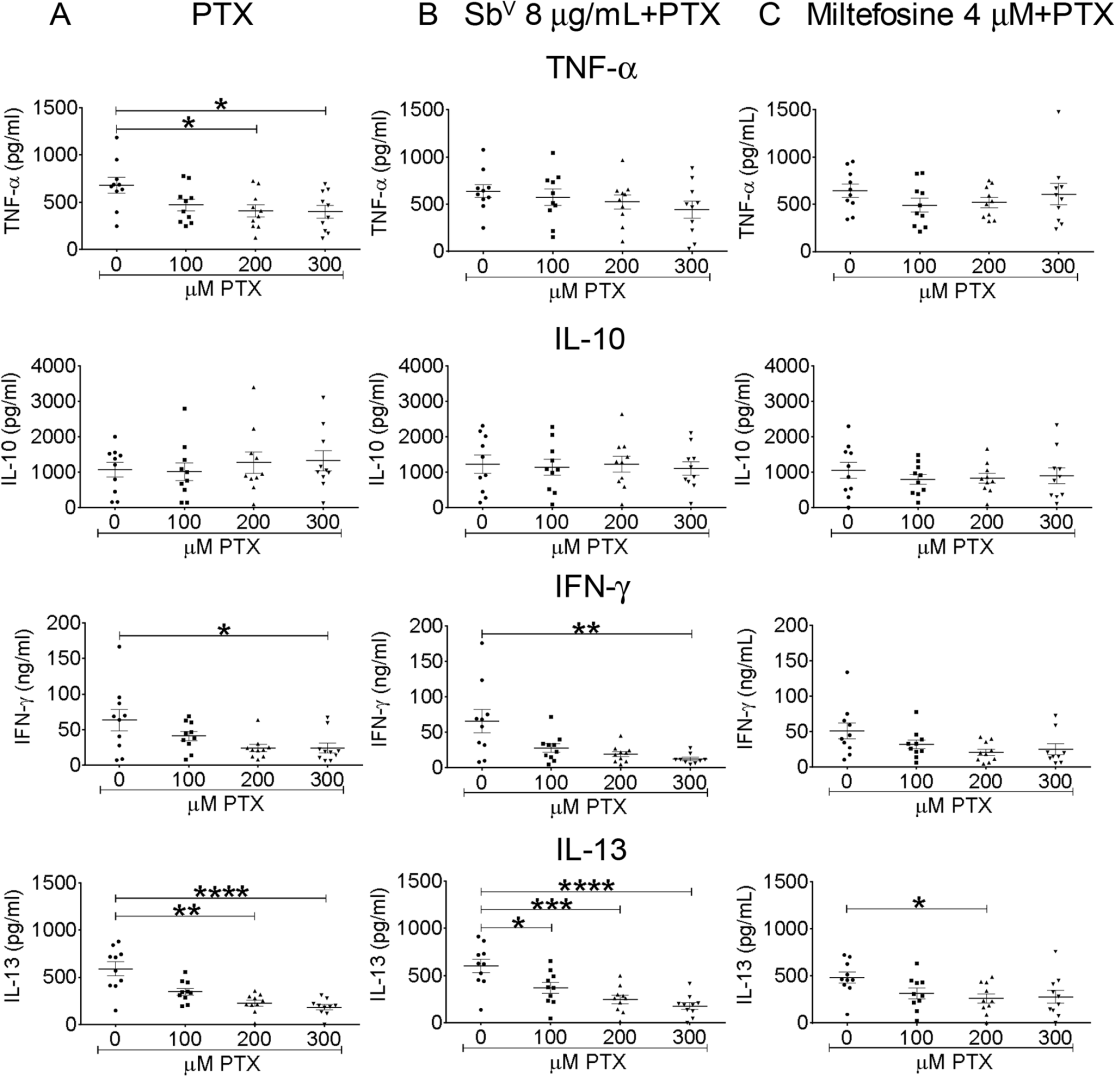
Dose-response of cytokine secretion by PBMCs to pentoxifylline and anti-leishmanial-pentoxifylline combinations. Cells from 10 patients were infected at a parasite: monocyte ratio of 10:1. Cytokine levels (IL-10, IL-13, TNF-α, IFN-γ) were determined after exposure for 96 h to different concentrations of pentoxifylline (PTX), (A) alone, combined with (B) 8 μg Sb^V^/ml meglumine antimoniate or (C) 4 μM miltefosine. Data are expressed as mean ± SEM. * p < 0.05, ** p < 0.01, *** p < 0.0001, **** p ≤ 0.0001.

A significant, dose-dependent reduction of TNF-α, IL-13 and IFN-γ secretion occurred when infected PBMCs were exposed to pentoxifylline (Fig 5A). The suppressive effect of pentoxifylline on IL-13 and IFN-γ production was conserved when this immunomodulator was combined with miltefosine or meglumine antimoniate (Fig 5B and 5C). Neither pentoxifylline alone or in combination with anti-leishmanial drugs affected the secretion of IL-10 induced by infection (Fig 5). Pentoxifylline did not induce the secretion of cytokines in uninfected PBMCs being either undetectable or present in amounts less than or equal to that detected in the corresponding control cultures without pentoxifylline.

**Fig 6:**
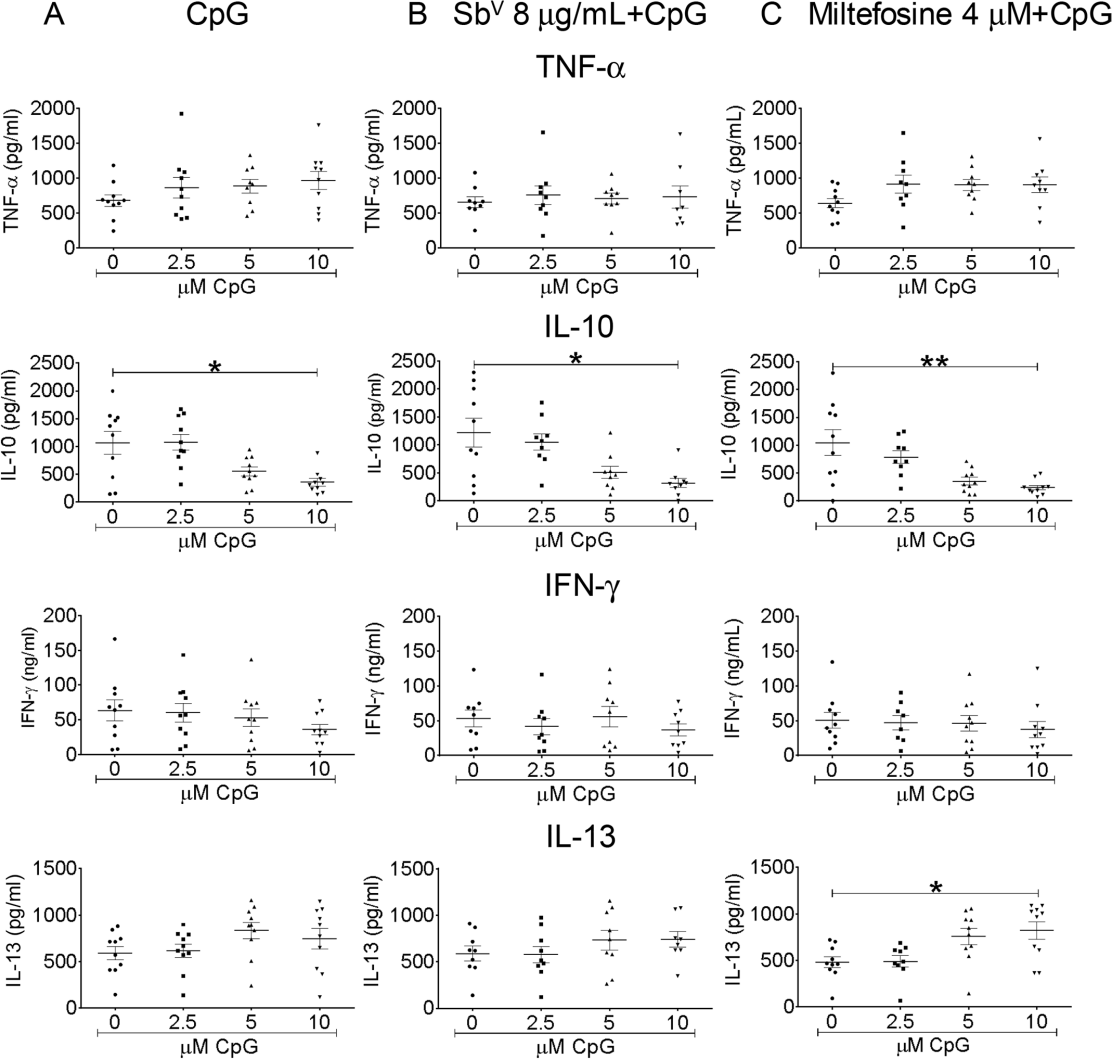
Dose-response of cytokine secretion by PBMCs to CpG, and anti-leishmanial- CpG combinations. Cells from 10 patients were infected at a parasite: monocyte ratio of 10:1. Cytokine levels (IL-10, IL-13, TNF-α, IFN-γ) were determined after exposure for 96 h to different concentrations of CpG (A) alone, combined with (B) 8 μg Sb^V^/ml meglumine antimoniate or (C) 4 μM miltefosine. Data are expressed as mean ± SEM. * p < 0.05, ** p < 0.01.

In the case of CpG, IL-10 secretion diminished significantly in response to this immunomodulator alone and in combination with miltefosine or meglumine antimoniate, whereas secretion of IL-13 increased in cultures exposed to the combination of miltefosine with CpG, reaching significance at 10 μM CpG (Fig 6). Neither TNFα nor IFN-γ secretion was altered by CpG or combinations of this immunomodulator with miltefosine (Fig 6A and 6C), nor was secretion of IL13, IFN-γ and TNF-α significantly altered by CpG in combination with meglumine antimoniate (Fig 6B). In uninfected PBMCs, CpG induced the secretion of IL-10 (median concentration for PBMCs without stimulus was 0 vs 151 pg/mL with 10 μM CpG; Mann Whitney test *p = 0*.*0023*, n = 8) but did not induce secretion of IL-13 (median without stimulus, 38 pg/mL vs 42 pg/mL with 10 μM CpG, Mann Whitney test *p = 0*.*2788*, n = 8) or TNF-α and IFN-γ, which were not detected in supernatants of uninfected PBMCs with or without CpG.

## Discussion

Pre-clinical assessment of alternative therapeutic approaches including drug combinations and co-adjuvant immunotherapy have been constrained by the unavailability of *in vitro* models that approximate the *in vivo* response. The results of this investigation substantiate the feasibility of an integrated evaluation of parasite viability and host response using an *ex vivo* model of infection based on peripheral blood mononuclear cells and *Leishmania* transfected with a luciferase reporter gene construct. This innovative strategy allows anti-parasitic efficacy and immunomodulatory effects of anti-leishmanial drugs and immunotherapeutic agents to be determined in systemically circulating cells of the human host, and thereby, access to the interplay of the antimicrobial agent and innate and acquired host defense.

Parasites transfected with reporter genes have been successfully employed to quantify parasite survival *in vitro*, *in vivo* and *ex vivo* [34, 41]. In particular, experiments that evaluated the susceptibility of intracellular amastigotes to meglumine antimoniate and miltefosine using luciferase activity as a measure of parasite viability have substantiated high correlation with conventional microscopy [38, 39]. In this *ex vivo* model, the use of luciferase transfected *Leishmania* allowed us to quantify viable parasites in host macrophages differentiated during culture of human mononuclear cells (lymphocytes and monocytes) exposed to live promastigotes, and to do so without the biases inherent to visual assessment by microscopy. Importantly, assay of primary monocyte-derived macrophage infection was achieved in a 96-well plate format as utilized for assessment of cell-mediated immune responses using PBMCs. The advantages of microcultures of human cells are further extended by multiplex assays which require minimal volumes of supernatant (25–50 μl) for the quantification of cytokines and other mediators. Cytometric (FACS) analyses of cell subsets and gene expression assay are also feasible using cells from these microcultures, thereby broadly expanding the immunological information that can be accessed using this analytic approach.

The robustness of the assessment of parasite burden in PBMCs was supported by readily measurable signal for both 10:1 and 20:1 parasite to cell ratios at all time intervals evaluated over 3 days of culture post-infection and the comparable parasite burden of total PBMCs and adherent cells alone (Fig 1A). Furthermore, PBMC cultures allowed the simultaneous assessment of surviving parasites and secretion of multiple cytokines post-infection. Interestingly, although the parasite burden was proportional to the inoculum of promastigotes, cytokine secretion was not (Fig 1A and 1B). The independence of these parameters was also shown in the divergence of cytokine responses and parasite survival in the presence of increasing concentrations of meglumine antimoniate and miltefosine (Fig 2), in the distinct kinetics of these responses during exposure to a single concentration of antileishmanial drug (Fig 3), and is generally supported by other studies [42, 43].

The conventional macrophage-amastigote model of drug susceptibility assessment evaluates the leishmanicidal effect of drugs that act directly on the parasite or the host macrophage. This long and widely used approach is not informative about collateral immunomodulatory effects of these drugs or for agents whose activity is mediated by other cells of the immune system. Hence, neither the effect of the immune response on the outcome of treatment nor the anti-leishmanial drug on the immune response is evaluable with macrophages alone. Importantly, in the *ex vivo* PBMC model, parasite viability diminished in a drug concentration-dependent manner, as occurs in the macrophage-amastigote model of drug susceptibility evaluation [34, 44].

Notably, at the concentrations evaluated in combination with immunomodulators, and which are below their CMax *in vivo* [45, 46], neither meglumine antimoniate nor miltefosine altered cytokine expression of uninfected or infected PBMCs (Fig 3). Hence, these drug concentrations allowed assessment of immunodulation by CpG and pentoxifylline without the confounding influence of the antileishmanial drugs. Although time-dependent reduction in parasite viability occurred in the absence of treatment, parasite burden was sustained and significantly higher than in treated cultures from 48 hours onward. This attrition of infection is likely mediated by the activation of effector mechanisms in sensitized lymphocytes and host macrophages.

Although miltefosine has been reported to induce IFN-γ secretion in uninfected human mononuclear cells [47], in the corresponding study, an approximately 5-fold higher concentration of miltefosine (19.6 μM versus 4 μM) was utilized and included co-stimulation with either IL-2 or IL-2/phytohemagglutinin [47]. We did not observe induction of IFN-γ secretion in dose response experiments with miltefosine even at comparable or higher concentrations of miltefosine (Fig 2) but secretion of IL-10 and IL-13 was significantly reduced in a dose dependent manner. Miltefosine has been shown to act in multiple ways and is an AKT (serine, threonine kinase) inhibitor, which is known to be involved in immune activation [48]. However the specific effects of miltefosine on the immune system are not well understood. The suppression of IL-10 and IL-13 secretion by miltefosine in PBMCs infected with *Leishmania* is a novel finding. Attribution of suppression of these cytokines to miltefosine is supported by the disparity between the dose-response of the parasiticidal and immunomodulatory effects of this drug as well as the absence of immunomodulatory effects of meglumine antimony despite the significant dose-dependent reduction of parasite burden (Fig 2).

In this study we found that compounds that modify the host immune response can affect the parasiticidal activity of anti-leishmanial drugs, as evidenced by a significant reduction in parasite killing when meglumine antimoniate was combined with CpG. The precise mechanisms, remain to be determined and will be of interest for future studies to define the interactions and “cross-talk” between drug-regulated and immunomodulatory processes. Considering the interdependent relationship between the host immune response and the efficacy of anti-leishmanial treatment, as illustrated by the high incidence of failures and relapses in immunocompromised patients [49–51], this model approximates natural infection and is particularly useful for the study of therapeutic interventions involving immunomodulation.

The contribution of immune response to the efficacy of anti-leishmanial treatments is acknowledged yet poorly understood. Several *in vivo* studies suggest a direct relation between the leishmanicidal effect of antimony and host immune factors like IFN-γ, TNF-α and IL-12 [52–55], and recent evidence suggests that activation of the host immune response by miltefosine is a constituent of its mechanism of action [56–58]. The inclusion of sensitized cells of the adaptive immune response in the assessment of therapeutic agents allowed the quantification of pro-inflammatory, anti-inflammatory and Th1/Th2 cytokines that are elicited over the clinical spectrum of infection by species of the *Viannia* subgenus [59–61]. Further, high expression of pro-inflammatory chemokines such as CCL2, CXCL-9 and CXCL-10 has been associated with chronicity of dermal leishmaniasis caused by *L*. *(Viannia) braziliensis* infection [48]. Reactive oxygen species and nitric oxide production have also been identified among effector mechanisms that may be induced by antimonial drugs and participate in the elimination of *Leishmania* [62]. These and other potential immune response parameters including cell phenotypes and gene expression associated with clinical outcome and therapeutic response can also be assessed using this model.

Immunomodulators of potential use in the treatment of cutaneous leishmaniasis, demonstrated concentration dependent effects critical to the outcome of *Leishmania* infection and disease. These *ex vivo* results confirm the usefulness of PBMCs to determine whether interventions that modify the host immune response can affect parasite survival and to what extent. The observed suppressive effect of pentoxifylline on cytokine production was consistent with previous reports using re-stimulation of PBMCs with soluble *Leishmania* antigen and *in vitro* immune responses during other pathologies associated with exacerbated inflammatory response [63–66].

The relevance of these findings to treatment is also illustrated by the recently reported results of a pilot study in cutaneous leishmaniasis patients infected with *L*. *(V) braziliensis* treated with the combination of antimony and pentoxifylline [21]. In latter study, both the conventional and combination treatments were accompanied by reduced secretion of pro-inflammatory TNF-α and IFN-γ by re-stimulated PBMCs on day 15 of treatment compared to pretreatment. However, the reduction was more pronounced in the antimony plus pentoxifylline group (84% vs 48%). In the *ex vivo* analysis of PBMCs from patients infected with *L*. *(V) panamensis*, the combination of meglumine antimoniate and pentoxifylline resulted in a significant reduction in secretion of IFN-γ and the Th2 cytokine IL-13.

CpG alone significantly induced the secretion of IL-10 in uninfected PBMCs, corroborating a previous report [67], yet interestingly, CpG in combination with antileishmanial drugs diminished the secretion of IL-10 by infected-PBMCs. Similarly the decrease of IL-10 and TGF-β by CpG2006 alone or in combination with miltefosine was reported recently by Shivahare and collaborators in the models of *L*. *donovani* infection in hamster and BALB/c mice. Therefore the use of immunomodulatory agents together with anti-leishmanial drugs may yield effects that differ from what has been previously observed for the agents alone, underscoring the importance of pre-clinical evaluation of immunotherapeutic strategies.

In conclusion, the *ex vivo* PBMC model of *Leishmania* infection provides access to host as well as parasite determinants of therapeutic response. Analysis of the interplay of acquired and innate immunity and anti-leishmanial drugs and co-adjuvant immunotherapeutic agents is achievable using PBMCs from donors previously exposed to natural infection with *Leishmania*. Using this approach, therapeutic strategies for leishmaniasis, particularly those that seek to intervene in the immune and inflammatory response can be evaluated and adjusted to optimize host and parasite responses to achieve healing.
